# Mobile apps, AI, and teletherapy: a comprehensive review of digital mental health tools for nurses

**DOI:** 10.3389/fpubh.2025.1686766

**Published:** 2026-01-05

**Authors:** Weiwei Huang, Yan Xing, Feng Zhao, Yingying Wang

**Affiliations:** 1 Department of Neurosurgery, China-Japan Union Hospital of Jilin University, Changchun, China; 2 Second Inpatient Area of Urology Department, China-Japan Union Hospital of Jilin University, Changchun, China; 3 Department of Operating Room, China-Japan Union Hospital of Jilin University, Changchun, China; 4 Department of Thyroid Surgery, China-Japan Union Hospital of Jilin University, Changchun, China

**Keywords:** nurses, burnout, digital mental health interventions, mobile mental health apps, teletherapy, accessibility

## Abstract

Chronic understaffing, workplace violence, moral distress, rotating shifts, and administrative burdens have created a global mental health crisis for nurses. Around half to two-thirds of nurses report symptoms of burnout, and large surveys have found high levels of depression and anxiety among nursing staff. The COVID-19 pandemic exacerbated these issues, increasing absenteeism, turnover, and error rates. Barriers to care—such as stigma, cost, and limited access in rural areas—mean that many nurses remain untreated. Digital mental health interventions (DMHIs) offer scalable, flexible, and often anonymous support tailored to nurses’ schedules and risks. These include teletherapy platforms, AI-driven chatbots and support systems, mobile mental health apps, and hybrid digital-human models. Recent studies (2020–2025) suggest DMHIs can reduce anxiety, depression, and burnout while improving resilience, job satisfaction, and retention. However, obstacles such as unequal access, variable digital literacy, privacy concerns, and limited long-term evidence slow adoption. This review synthesizes current research on DMHI types and efficacy, and examines factors affecting their accessibility and integration into nursing practice. We also discuss cultural and ethical considerations and strategies for involving nurses in designing these tools. Our analysis identifies gaps and opportunities for developing nurse-centered digital mental health solutions that strengthen the workforce and improve patient care.

## 1 Introduction

As the nursing profession experiences unprecedented levels of psychological distress, the mental health of nurses has become a global concern. Nurses, the foundation of health care systems, manage the psychological and physical demands of their jobs while providing critical clinical care. However, their mental health is continually challenged by systemic issues such as high patient-to-nurse ratios, staffing shortages, irregular schedules, exposure to traumatic events, and increased administrative demands. It is concerning how common mental health problems are among nurses. An estimated 11.23–70% (1) of nurses worldwide experience burnout, 35% (2) experience depression, and up to 30% (3) report having post-traumatic stress disorder (PTSD) in high-stress specialties like emergency medicine, psychiatric nursing, and critical care. The way healthcare is delivered is directly impacted by these conditions. Emotional exhaustion and burnout are associated with higher turnover rates, lower patient satisfaction, and more medical errors, all of which worsen staffing shortages and workload burdens (2).

These strains were exacerbated by the COVID-19 pandemic, which added new difficulties and heightened preexisting stressors. Long-term exposure to critically ill patients, quickly changing clinical procedures, and moral distress from circumstances like giving end-of-life care without family presence were all challenges faced by frontline nurses (4, 5). Insomnia, anxiety, and depression reports increased significantly (6), and many nurses thought about quitting nursing altogether (7, 8).

Even though these problems are becoming more widely acknowledged, there are still major obstacles in the way of receiving quality assistance. Stigma is still widespread: more than 36% (9) of nurses steer clear of mental health services out of concern for disclosure, damage to their reputation, or consequences to their licensure. Inequities are made worse by financial limitations and a lack of services, especially in rural or low-resource areas (10, 11). The acute stress profiles and erratic schedules of nursing work are frequently not accommodated by traditional mental health care models (12).

Digital mental health interventions (DMHIs) have become a promising tool to address these issues in this context. DMHIs use technology to offer 24/7, user-specific mental health support that can transcend time and location constraints (13, 14). These interventions cover a variety of approaches, such as:

Applications for mobile mental health that provide stress management tools, mood monitoring, cognitive behavioral therapy (CBT) exercises, and mindfulness training (1, 2, 15). AI-driven support systems, like chatbots that can provide individualized self-help materials, early distress detection, and real-time emotional support (16). Platforms for virtual counseling and teletherapy allow for synchronous or asynchronous communication with certified mental health specialists (17, 18). Hybrid models, which combine human-led care with digital resources to improve follow-up and personalization (19, 20).

These solutions’ scalability is especially important given the state of healthcare today, where access to in-person mental health services is restricted due to a lack of staff. Recent research shows that DMHIs are effective in helping nurses feel less stressed, depressed, and anxious while also improving their coping mechanisms and resilience (14, 21). AI-driven chatbots have demonstrated notable improvements in mood and self-care engagement (16), while mindfulness-based mobile applications have been demonstrated to reduce perceived stress by as much as 30% (1, 2). However, there are obstacles to DMHI adoption and sustainability. Nurses’ levels of digital literacy vary, and senior staff frequently need extra instruction to use these tools efficiently (2, 22). Inequitable implementation is hampered by the digital divide, which is fueled by differences in internet access and device availability, especially in underserved or rural areas (10, 11).

Concerns about data security and privacy are also very important, particularly as AI systems interact with electronic health records (EHRs) (23, 24). Furthermore, if algorithmic bias in AI-driven DMHIs is not addressed, it may inadvertently perpetuate health disparities (25). A multifaceted strategy is needed to address these issues. In order to guarantee relevance, usability, and cultural competence, nurses should be directly involved in the design of DMHI, which should be guided by user-centered and participatory design principles (24, 26). Second, to protect user trust, ethical AI frameworks and robust privacy protocols must be integrated (9, 18). Third, equitable adoption requires focused approaches to close the digital divide, like subsidizing devices, enhancing rural connectivity, and providing specialized training in digital literacy (11, 22).

The goal of this review is to present a thorough synthesis of the available data supporting DMHIs in nursing practice. Using works of literature from 2020 to 2025, it will:

1. List the main DMHI categories along with their primary functions.
2. Assess their effectiveness in treating PTSD, anxiety, depression, and burnout in nurses.
3. Analyze the elements that affect accessibility, usability, and integration in clinical and professional contexts.
4. Talk about ethical issues such as bias, privacy, security, and striking a balance between technology and interpersonal relationships.
5. Determine areas for innovation and research gaps.

The review aims to educate nursing professionals, policymakers, and healthcare leaders on how to successfully implement and maintain DMHIs as a component of a larger mental health strategy by examining these dimensions. The ultimate objective is to use digital innovation to create resilient, supported nursing workforces, which will enhance patient care quality and safety while also enhancing nurse well-being.

## 2 Methods

This narrative review synthesizes recent evidence on digital mental health interventions (DMHIs) for nurses, drawing on systematic search and selection processes to ensure rigor and relevance. The approach combines narrative synthesis with elements of systematic methodology to classify DMHIs, evaluate their efficacy, and explore implementation factors, aligning with the review’s objectives outlined in the Introduction.

### 2.1 Literature search strategy

A systematic literature search was conducted across databases selected for their coverage of nursing, mental health, and digital health topics, including PubMed, CINAHL, PsycINFO, Scopus, and Web of Science. These databases were chosen due to their extensive indexing of peer-reviewed articles in healthcare, psychology, and technology domains relevant to nursing practice. The search was limited to articles published between January 2020 and November 2025 to focus on post-COVID-19 developments and recent technological advancements in DMHIs, capturing the pandemic’s impact on nurse mental health and the rapid evolution of digital tools. Keywords and Boolean operators were used to identify pertinent studies, with example search strings including: “nurses AND burnout AND digital interventions”; “nurses AND (depression OR anxiety OR PTSD) AND (mobile apps OR AI OR teletherapy)”; “digital mental health AND nursing AND (accessibility OR usability)”; and “AI-powered support AND nurses AND ethics.” Variations in terminology (e.g., “mental health apps” OR “telehealth”) were incorporated to broaden capture while maintaining specificity.

### 2.2 Inclusion and exclusion criteria

Studies were included if they were peer-reviewed articles in English, published within the 2020–2025 timeframe, and focused on DMHIs (e.g., mobile apps, AI tools, teletherapy, or hybrid models) applied to nurses’ mental health outcomes, such as burnout, depression, anxiety, PTSD, resilience, or job satisfaction. Empirical studies (e.g., randomized controlled trials, meta-analyses, qualitative studies) and systematic reviews were prioritized for their evidence-based insights, with relevance to nursing populations (e.g., hospital, critical care, or rural settings) as a key criterion. Exclusions encompassed non-empirical works (e.g., editorials, commentaries without data), studies unrelated to mental health outcomes or DMHIs, those not specific to nurses (e.g., general healthcare workers without nurse subgroups), and articles in languages other than English or outside the specified time frame.

### 2.3 Selection and screening process

The initial search yielded approximately 1,200 records. After removing duplicates (*n* ≈ 400), 800 unique records remained for title and abstract screening. Full-text review was conducted on 450 articles meeting preliminary criteria, resulting in 228 studies included for synthesis (as cited in the References). Screening followed a multi-stage process inspired by PRISMA guidelines: initial title/abstract review for relevance, followed by full-text assessment for alignment with inclusion criteria. Inter-rater agreement was achieved through discussion among authors where discrepancies arose, ensuring consistency. This process mitigated selection bias by prioritizing comprehensive coverage of global and specialty-specific evidence while excluding low-relevance sources.

## 3 Issues with mental wellness nurses’ perspective

Occupational stressors, a high prevalence of psychological disorders, and obstacles to receiving support make mental health issues for nurses around the world severe. We need creative digital solutions to address these issues and enhance healthcare delivery. Burnout rates of 11.23–70%, depression of up to 35.1%, and PTSD prevalence of up to 30% in high-stress environments, especially during the COVID-19 pandemic, are caused by chronic understaffing, workplace violence, moral distress, rotating shift work, and administrative burdens (1, 3, 6, 27–29). Hospitals spend about $16,736 per nurse annually as a result of these problems, which are worsened by specialty-specific demands (such as ICU and psychiatric nursing) and systemic factors like staffing shortages. Major repercussions result from these problems, including 20–30% (27, 30–32) nurse turnover rates and a 10% (27) increase in patient care errors. Support uptake is further hampered by stigma, more than 36% (9) of nurses avoid care out of fear of professional consequences, as well as financial limitations and access inequalities, especially in rural and low-resource settings (9, 33). Digital mental health technologies (DMHTs), including teletherapy, AI-powered tools, and mobile apps, present promising solutions by offering 24/7 accessible, scalable, and anonymous interventions that are customized to nurses’ specific needs, such as shift-based schedules and trauma exposure (13, 14). The factors that lead to mental health issues among nurses, their frequency and effects, obstacles to getting help, and how digital solutions can improve patient safety, nurse retention, and the effectiveness of the healthcare system are all covered in this section.

### 3.1 Stressors at work

Numerous work-related stressors that nurses face have a substantial impact on mental health issues, which illustrates the importance of digital interventions that are scalable and nurse-focused. Chronic understaffing is linked to a 62% global burnout prevalence (ICN, 2023) and a 10% increase in patient care errors because of increased workload and emotional exhaustion (27). In certain acute care settings, nurse-to-patient ratios can reach 1:10. This burden is further compounded by workplace violence, as nurses who experience verbal or physical aggression are two to four times more likely to report burnout, anxiety, and post-traumatic stress disorder (PTSD) (28, 34). Due to wasteful treatment, a lack of resources, and moral conundrums like caring for patients who are dying without family present, the COVID-19 pandemic exacerbated moral distress among intensive care unit (ICU) nurses (4, 5). Due to the disruption of circadian rhythms caused by rotating shift work, depression and insomnia are more common, especially among young female nurses who are experiencing social jetlag (35, 36). Many nurses spend more than two hours a day on excessive administrative duties, especially electronic health record (EHR) documentation, which is associated with a moderate risk of burnout and higher intentions to leave the profession (37, 38). Role overload and organizational policies cause acute stress for intensive care unit nurses, and nurses in public hospitals report higher levels of exhaustion than those in private settings (39, 40). These stressors, when combined with systemic problems like stigma and staffing shortages, points to the urgent need for digital mental health tools like AI-driven stress monitoring, teletherapy for trauma support, and mindfulness mobile apps. These tools offer 24/7 access, anonymity, and customized interventions to reduce occupational stress and improve resilience.

### 3.2 Impact and prevalence

**Figure 1:**
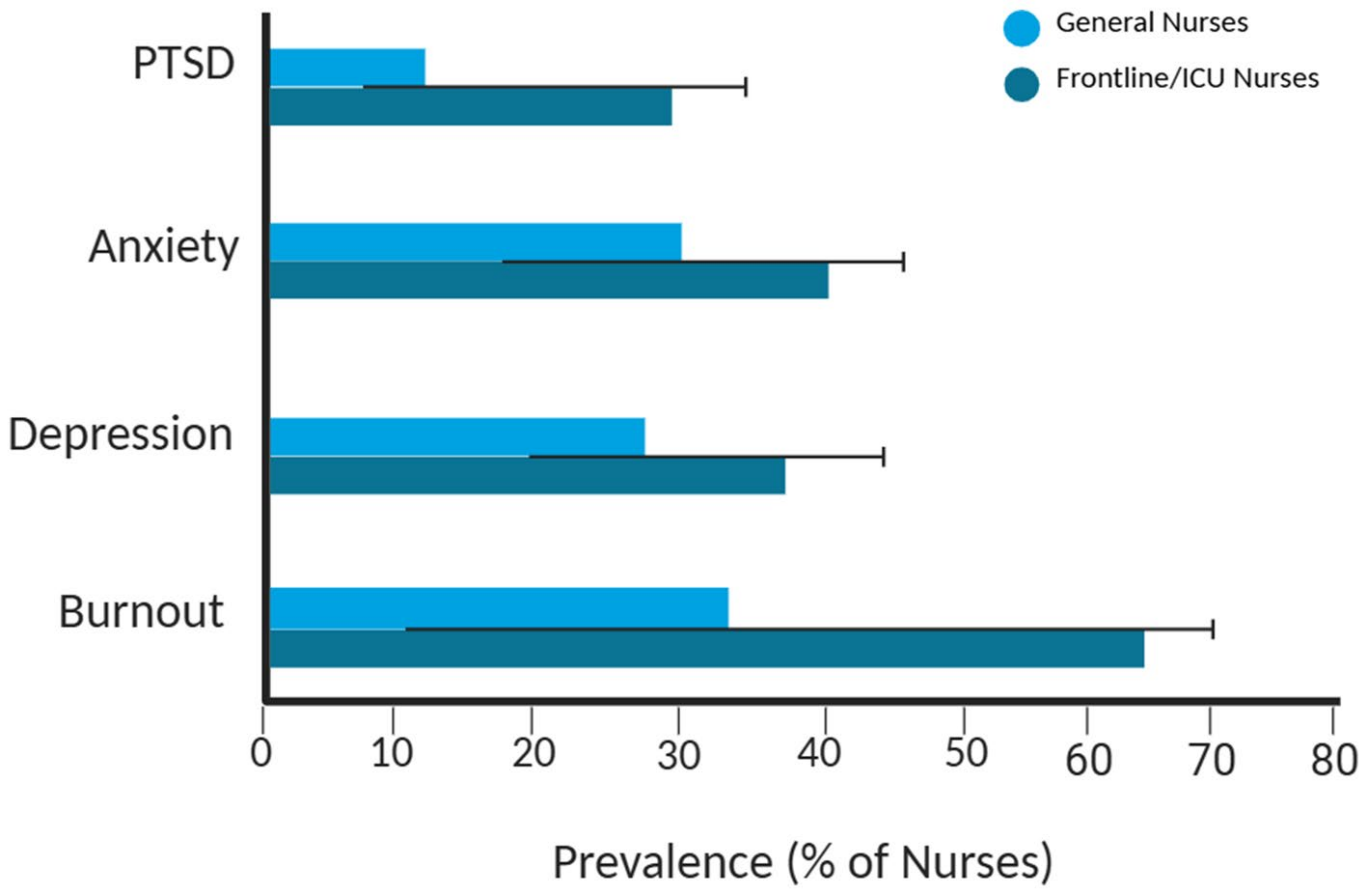
Prevalence of mental-health conditions among nurses (2020–2025). The figure illustrates the average and observed range of burnout, depression, anxiety, and post-traumatic stress disorder (PTSD) among nurses across different care settings. Lighter bars represent the general nursing population, while darker bars indicate ICU and frontline nurses. Black lines denote reported minimum and maximum prevalence values. The data highlight the higher mental-health burden observed among nurses working in high-intensity and pandemic-exposed environments.

Nurse retention, patient safety, and the effectiveness of the healthcare system are all greatly impacted by the global mental health crisis among nurses, which is marked by high rates of burnout, depression, and PTSD. This highlights the necessity of digital mental health interventions. The prevalence of burnout varies between 11.23 and 70% (1) worldwide, and sub-Saharan African nurses report higher rates of depersonalization (60%) and emotional exhaustion (66%) (1, 40). Up to 35.1% of nurses suffer from depression, which has a moderate relationship with burnout (r = 0.403). It is worsened by stress at work and is impacted by how nurses express their anger, especially in the early stages of their careers (2, 41). In high-stress environments, like conflict-affected areas of sub-Saharan Africa, the prevalence of PTSD can reach 30% (2). It has also increased dramatically since COVID-19, with psychological distress in China rising from 27.7% before the pandemic to 57.6% by 2024 (6). There are clear differences by specialty: psychiatric nurses report higher levels of stress, while intensive care unit nurses have lower levels of compassion satisfaction and burnout than oncology nurses (30, 42). These difficulties were exacerbated by the COVID-19 pandemic, as frontline nurses showed higher levels of moral distress, depression, and insomnia, which led to more people wanting to quit their jobs (7, 8). Burnout has a substantial financial impact; hospitals lose $16,736 per nurse each year as a result of turnover, which could be reduced to $11,592 with focused interventions (31, 43). In addition to compromising patient outcomes because of errors and decreased satisfaction, non-economic effects include a 10% increase in emotional exhaustion over a one-year period, which results in a 12% increase in organizational turnover per unit increase on the emotional exhaustion scale (32, 44). Although methodological limitations in validation require context-specific refinements, validated screening tools like the Well-Being Index (WBI), SRQ-20, GHQ-12, and PHQ-9 effectively identify distress and stratify risks, including intent to leave and care errors (45–47). To lessen mental health burdens, cut down on turnover costs, and enhance patient care quality across a range of healthcare settings, these findings underscore the urgent need for easily accessible, nurse-tailored digital tools, such as teletherapy for PTSD, AI-driven risk detection, and mobile apps for mood tracking. As shown in Table 1, the prevalence of mental-health issues among nurses remains alarmingly high across global and specialty-specific populations. To provide a clearer visual overview of these disparities, Figure 1 illustrates the average and observed ranges of these conditions between 2020 and 2025 across different care settings.

**Table 1:** Prevalence of mental health issues among nurses across global and specialty-specific populations.

Condition	Global prevalence range (%)	Regional/specialty notes
Burnout	11.23–70	Up to 66% emotional exhaustion and 60% depersonalization in Sub-Saharan Africa; higher in ICU nurses (1, 29, 42)
Depression	20–35.1	Increased rates post-COVID, especially in frontline nurses (2, 7)
PTSD	8–30	27.7–57.6% among Chinese nurses pre−/post-COVID; ~30% in conflict zones (3, 6)
Anxiety	25–40	levated post-COVID among frontline nurses globally (7)
Psychological Distress	27–58	Markedly higher post-COVID (up to 57.6%) (6)

### 3.3 Obstacles to assistance

Significant obstacles prevent nurses from getting mental health care, which exacerbates psychological distress and demonstrates the potential for digital tools to offer anonymous, easily accessible, and reasonably priced solutions. A major obstacle is stigma; more than 36% (9) of nurses steer clear of mental health services out of fear of disclosure, shame, or professional consequences, including issues with licensure (9, 12, 33, 48). Personal, professional, and employment-related stigma is influenced by structural and cultural factors, such as nursing specialization and attitudes in the workplace, especially in high-stress environments like psychiatric care (49, 50). These obstacles were worsened by the COVID-19 pandemic, as rising rates of anxiety, PTSD, depression, and burnout discouraged people from seeking help because of increased demands at work (9). Despite the demonstrated effectiveness of employer-sponsored programs in reducing symptoms and producing positive returns on investment, many nurses are excluded due to financial constraints, such as high therapy costs and limited access to alternative-to-discipline programs for substance use disorders (51, 52). Disparities in access are particularly noticeable in rural areas, where care is hampered by a lack of mental health professionals, inadequate facilities, and a lack of anonymity, especially in low-resource nations like Kenya (10). Because of stigma and low mental health literacy, nursing students face particular challenges and frequently choose unofficial support from friends and family (12). Systemic disparities are highlighted in Europe, where differences in the use of mental health services are correlated with the availability of resources (such as the number of psychiatrists per capita) (53). Peer support programs like the Resilience In Stressful Events (RISE) program, which offers affordable stress management (net benefit: $22,576.05 per nurse) and consumer-led mentoring to lessen stigma, are examples of promising interventions (54, 55). Through 24/7 access, affordability, and privacy, digital mental health tools—such as AI-driven care navigation, teletherapy for anonymous counseling, and mobile apps with low-threshold interventions (like mindfulness and CBT-based exercises)—address these barriers and are in line with recommendations for early risk identification, peer support networks, and culturally appropriate interventions to increase nurses’ uptake of mental health support (12, 33, 56).

### 3.4 Digital solutions are necessary

By offering anonymous, scalable, and round-the-clock solutions that are customized to their occupational stressors and care-related barriers, DMHTs have the potential to significantly improve the mental health of nurses. Disparities in access, financial constraints, and stigma exacerbate high rates of burnout, depression, and PTSD, especially in rural areas [Sections 2.2, 2.3]. By providing anonymity, DMHTs help to overcome these obstacles and help the more than 36% of nurses who are discouraged from getting help because they fear stigma and professional consequences (9, 33). Studies have demonstrated that internet-delivered mindfulness-based stress reduction (iMBSR) is more effective than in-person programs in reducing depressive symptoms. Mobile apps, which are commonly used by nurses to promote health-promoting behaviors, offer simple, low-threshold interventions like mindfulness and CBT-based exercises (13, 15). Scalability is a major benefit since DMHTs improve quality of life and depressive symptoms while providing round-the-clock access, which is essential for nurses with erratic schedules and has demonstrated viability in low- and middle-income nations (14, 57). Social elements improved engagement, and eHealth and mHealth interventions proved safe and effective during the COVID-19 pandemic (58). For time-constrained nurses, nonclinician-guided digital interventions that use persuasive design elements, such as emails and text reminders, increase accessibility and adherence while producing results that are comparable to those of clinician-guided interventions (21, 59). To maximize adoption, however, obstacles like technical problems, a lack of personalization, and organizational hurdles (like inadequate training or workflow integration) call for person-centered designs and evidence-based training (22, 60). While integration with EHRs can expedite monitoring, it also runs the risk of increasing documentation burden, which could exacerbate burnout (61, 62). For nurses, DMHTs such as AI-driven CBT assistants and secure social networking tools address stigma and rural access gaps. Although more research is required to ensure cost-effectiveness and long-term efficacy, DMHTs promise to improve mental well-being, lower turnover (costing $16,736 per nurse annually), and improve patient care quality by providing scalable, anonymous, and customized interventions that meet nurses’ needs for flexible, stigma-free support (Section 2.2) (14, 63).

## 4 Digital mental health tool classification

**Figure 2:**
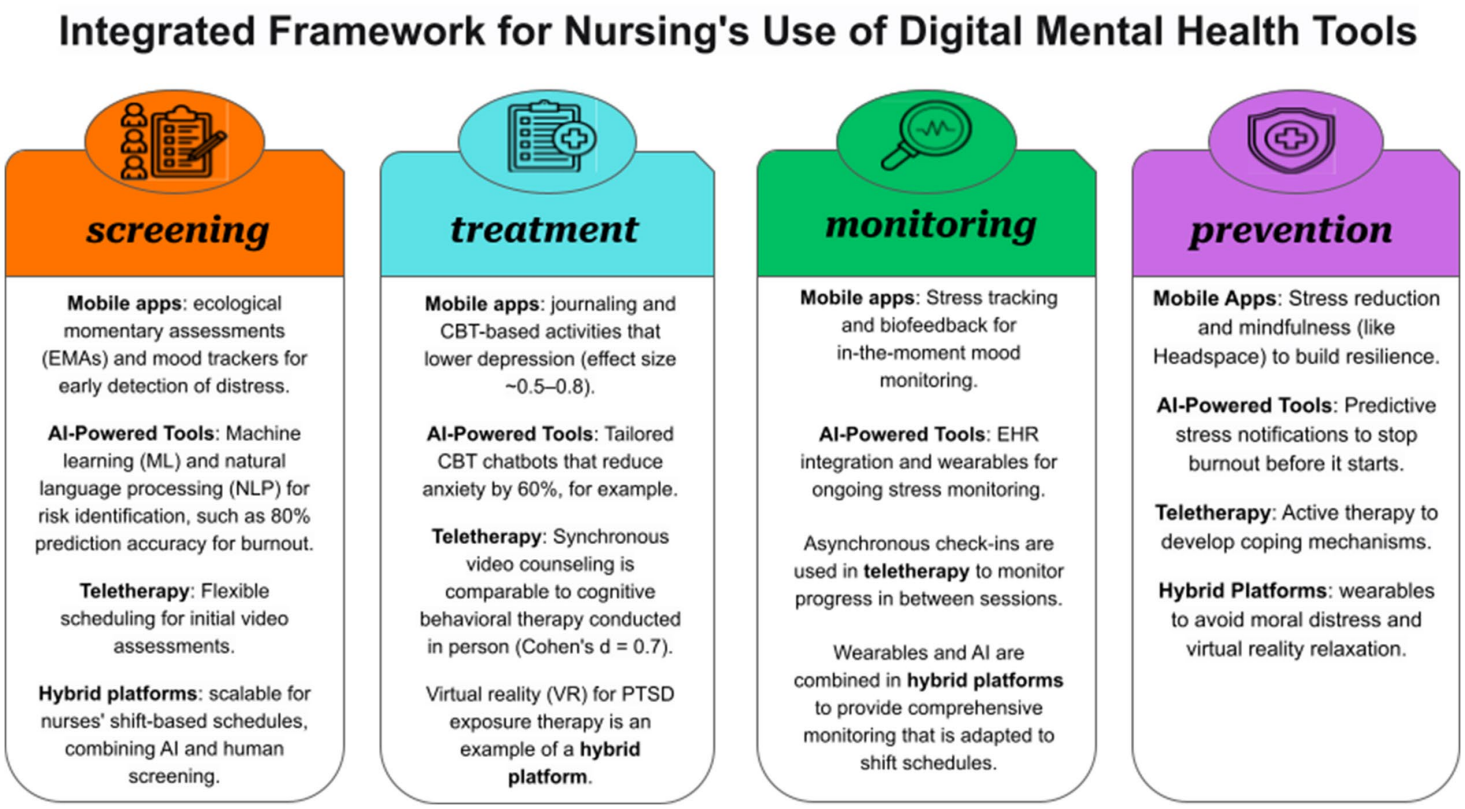
The integrated framework for digital mental health tools in nursing practice. This diagram shows a four-pillar model (screening, treatment, monitoring, and prevention) for digital mental health tools, teletherapy (e.g., BetterHelp, Talkspace), hybrid/emerging platforms (e.g., Ginger, VR, wearables), AI-powered tools (e.g., Wysa, ML-based burnout prediction), and mapping mobile apps (e.g., Headspace, NurseMind). In order to facilitate successful implementation in clinical settings, the framework emphasizes their roles in addressing stressors unique to nurses, such as shift work and trauma exposure.

As mentioned, digital technologies’ quick development has transformed mental health care for nurses by providing creative answers to their particular work-related stressors, such as shift work, exposure to trauma, and heavy workloads. Burnout is a global mental health crisis among nurses, with a prevalence of 50 to 70% (64, 65). Digital tools like teletherapy platforms, mobile applications, AI-powered systems, and hybrid interventions offer scalable, accessible, and frequently anonymous support. Given the stigma associated with traditional mental health care (40 percent of nurses avoid services because of career-related concerns) and time constraints, these tools are especially important (66). This section evaluates the features, applications, and applicability of digital mental health tools in relation to nursing practice by classifying them into four main categories: mobile mental health applications, AI-powered support systems, teletherapy and virtual counseling, and hybrid and emerging platforms. This classification emphasizes the potential of these tools to reduce burnout, anxiety, and depression while addressing issues like usability, privacy, and equitable access by combining evidence from studies published between 2020 and 2025 and including examples unique to nurses (16, 19, 67, 68). To help researchers, clinicians, and policymakers comprehend and maximize these tools for nursing practice, the following subsections offer a thorough taxonomy that is backed by a conceptual framework (Figure 2).

### 4.1 Apps for Mobile mental health

Since they provide scalable and accessible interventions that are suited to their particular stressors—such as shift work and trauma exposure—mobile mental health applications have become crucial for promoting nurses’ mental health. These apps fall into four categories: stress management tools, mood trackers, apps based on CBT, and mindfulness-based interventions (MBIs). Studies indicate that mindfulness apps, like Headspace and Calm, which offer guided meditations and breathing exercises, significantly reduce stress and burnout in nurses (e.g., MBIs reduce perceived stress by 30%) (64, 69). Although time constraints hindered engagement, a mindfulness-based app pilot study for nurses impacted by the COVID-19 pandemic revealed improved resilience and PTSD symptoms (70). CBT-based applications, such as Moodpath and Sanvello, offer structured exercises to treat depression and anxiety. Randomized controlled trials (RCTs) have shown modest but significant improvements in nurses’ depressive symptoms (effect size ~0.5, *p* < 0.01) (71, 72). Mood trackers, which frequently use ecological momentary assessments (EMAs), allow for real-time emotional state monitoring, minimizing recall bias and facilitating prompt interventions. However, participant fatigue presents a problem (73, 74). Features like shift-specific stress tracking are available through stress management apps like NurseMind and Resilience Hub (NHS), which have high acceptability (80% satisfaction) but low adherence (20–30% completion rates) because of the burden of technology (67, 75).

Important features increase the usefulness of these apps for nurses. Short shift breaks coincide with guided meditations, which usually last 5–10 min and encourage participation (76). Though effectiveness varies by user disposition, journaling features have been demonstrated to lessen mental distress in populations exposed to trauma and promote resilience through self-reflection (77, 78). Apps for biofeedback, especially those that use heart rate variability (HRV-BF), show promise in lowering stress and anxiety in frontline healthcare workers. The results are similar to those of in-person sessions (*p* = 0.004) (79, 80). EMAs offer real-time data for tailored interventions, but to allay nurses’ privacy concerns, strong data security is necessary (81). Despite incorporating shift-based scheduling and trauma-focused content, nurse-specific tools such as Resilience Hub struggle to maintain long-term engagement because of workplace demands (65, 82). These apps are effective in the short term, but the lack of longitudinal data and integration challenges suggest that it takes more study to maximize usability and guarantee cultural competence for a variety of nursing populations.

### 4.2 Support tools powered by AI

Support tools driven by artificial intelligence (AI) that make use of chatbots, natural language processing (NLP), and machine learning (ML) offer scalable, individualized solutions to nurses’ mental health issues. AI chatbots like Wysa and Youper, utilizing NLP and CBT techniques, reduce anxiety and burnout among healthcare workers by providing real-time, anonymous support. Improvements in symptom management have been reported, with a 60% reduction in anxiety, *p* = 0.004 (16, 83). These chatbots provide continuous data collection and personalized responses, addressing issues like stigma and provider shortages. However, ethical concerns, such as data privacy and a lack of emotional depth in comparison to human therapists, necessitate human oversight and strong safeguards (84). Early identification of emotional distress in nurses is made possible by NLP applications that analyze text from social media or clinical narratives to detect burnout indicators with high accuracy (80–93%) (85, 86). Although external validation is limited, machine learning models like Random Forests can predict burnout and turnover with up to 92% (87) accuracy by integrating wearable data (such as heart rate and sleep). Given their high-stress work environments and requirement for discrete, accessible interventions, nurses will deem these tools especially pertinent.

Personalized interventions and early distress detection are two uses for AI tools. Chatbots provide customized CBT-based workouts, and machine learning models that use wearable sensors—like Fitbits—achieve 96.63% accuracy in stress detection, boosting resilience through prompt interventions (88, 89). Federated learning frameworks, which predict nurse stress with over 90% (90) accuracy, enhance privacy by evaluating biomechanical data without jeopardizing sensitive information. Emerging trends include integrating AI with EHRs to improve early diagnosis, reduce burnout through enhanced clinical workflows, and monitor mental health in real time (91, 92). Nevertheless, obstacles include algorithmic bias, interoperability problems, and the requirement for AI literacy training to guarantee nurses’ efficient use (93, 94). To solve privacy issues and preserve confidence, ethical frameworks like algorithmic auditing and sophisticated encryption are essential (95). To ensure usability and cultural competency, AI tools for nurses must be co-designed to accommodate shift-based schedules and trauma exposure. Despite their potential, these tools need more study to confirm their long-term effectiveness and handle ethical issues so they can be responsibly incorporated into nursing practice.

### 4.3 Virtual counseling and teletherapy

For nurses’ mental health needs, teletherapy and virtual counseling are essential because they provide accessible, flexible support that can be adjusted to their hectic schedules and stigma-related issues. The modalities of these interventions are divided into synchronous (like BetterHelp’s video-based platforms) and asynchronous (like Talkspace’s text-based platforms). The effectiveness of synchronous text-based interventions is comparable to in-person counseling for anxiety and depression (*p* < 0.05), and they show notable improvements in mental health outcomes when compared to waitlist controls (17). Synchronous and asynchronous telepsychiatry both significantly reduced symptoms, according to a primary care RCT that found no significant differences in outcomes (96). In contrast to synchronous or in-person approaches, asynchronous modalities necessitate specific clinical skills, such as adjusting to text-based communication, despite their effectiveness (97). Despite the lack of specific research on nurses, these modalities provide valuable solutions tailored to their needs. Synchronous video sessions offer real-time support for acute stressors, while asynchronous options facilitate engagement during irregular shift breaks (98, 99).

Flexible scheduling, confidentiality, and integration with employee assistance programs (EAPs) are some advantages of teletherapy for nurses. Since 40% (66) of nurses say that time constraints prevent them from receiving mental health care, flexible scheduling that allows for shift work and access during non-traditional hours is crucial. Studies have shown that self-disclosure is higher in anonymous virtual settings, like those that use augmented reality masks or virtual human interviewers, than in identifiable assessments (*p* < 0.01), indicating that anonymity helps alleviate stigma (100, 101). With teletherapy-embedded EAPs demonstrating utilization rates comparable to traditional programs and enhancing nurse well-being during crises like the COVID-19 pandemic, integration with EAPs improves accessibility (102). Tele-counseling has been used in hospital-sponsored programs, like the Cleveland Clinic’s wellness initiatives, to lower staff anxiety related to the coronavirus, with notable decreases in perceived risk (*p* = 0.03) (103). Nevertheless, there are drawbacks, such as a 10% (97) decrease in patient satisfaction ratings when compared to synchronous therapy and the requirement for clinician training to guarantee competency (97, 99). Although there is still a gap in long-term sustainability, programs that combine teletherapy with on-site psychological first aid demonstrate that sustained implementation necessitates leadership engagement and technology-driven accessibility (104, 105). To guarantee fair access in a variety of contexts and optimize teletherapy for stressors unique to nurses, more research is required.

### 4.4 New and hybrid platforms

Promising approaches to addressing nurses’ mental health issues in high-stress settings include hybrid and emerging platforms that integrate wearable technology, virtual reality (VR), and AI-driven tools with human intervention. By combining AI’s scalability with human empathy, hybrid models—like those that integrate AI chatbots with human therapists (such as Ginger)—improve therapy results and diagnostic accuracy. For mild-to-moderate depression, they are moderately effective, with effect sizes ~0.6, *p* < 0.05, which is comparable to low-intensity clinician treatments (19, 20). By offering scheduled human therapy for more complex problems and real-time chatbot support for instant stress relief, these platforms help nurses and lower dropout rates by 20% (106) when compared to stand-alone digital interventions. Although there are few studies specifically focusing on nurses, virtual reality exposure therapy (VRET) has shown a strong ability to reduce PTSD symptoms in populations exposed to trauma. Meta-analyses have reported moderate effect sizes (d = 0.7) and sustained benefits at 6-month follow-ups (107, 108). Using machine learning algorithms, wearable devices that use biosensors such as heart rate variability (HRV) and electrodermal activity allow for accurate stress detection in nurses with accuracies ranging from 80.4 to 99% (109, 110). For instance, HRV-based wearables provide real-time monitoring to avoid burnout by correlating stress levels with shift patterns (111).

With features designed specifically to address shift-based stressors and trauma exposure, these platforms are extremely pertinent to nurses. Given that 66% (112) of young adults switch from digital to in-person care when hybrid options include professional referrals, hybrid systems offer flexible, anonymous support that fits with nurses’ erratic schedules and concerns about stigma. For night-shift nurses who have disturbed sleep patterns, wearables that incorporate circadian rhythm-focused interventions—like Fitbit’s stress score—are essential (113). Tools that are trauma-informed, such as psychosocial screening apps for pediatric care or graphic narrative aids for emergency nurses, improve patient-centered care and resilience while lowering moral distress (114–116). But there are obstacles like algorithmic bias, cultural mismatches, and privacy issues; 25% of digital health apps do not have explicit data policies (20, 117). Adoption is made more difficult by engagement concerns, such as nurses’ inclination for interactive content rather than static and the requirement for co-design to guarantee usability (26, 118). In addition to strong ethical frameworks (such as GDPR-compliant encryption) that address privacy and bias, future development should place a high priority on nurse involvement in the design of trauma-informed, shift-compatible tools. Although more research is needed to confirm these platforms’ long-term effectiveness and guarantee their fair application in various clinical settings, they have the potential to significantly improve nurse mental health.

## 5 Assessment of online resources for mental health

In high-stress environments, such as hospitals, DMHIs are now essential for supporting the mental health of nurses and other healthcare professionals. These interventions, which include teletherapy, artificial intelligence (AI)-powered tools, and mobile apps, are intended to treat common conditions like burnout, anxiety, and depression. In order to enable equitable and efficient mental healthcare, this review evaluates the efficacy, usability, privacy, security, and accessibility of DMHIs, with a focus on nurses’ use of these tools. It also identifies areas for improvement.

### 5.1 Review of efficacy and evidence

It has been demonstrated that DMHI is moderately to highly effective in assisting nurses with their mental health issues. Stress, anxiety, and depression can be alleviated by mobile apps that employ mindfulness-based interventions and cognitive-behavioral therapy (CBT) (119, 120). The effect sizes are between 0.5 and 0.8 (*p* < 0.01). For instance, meta-analyses reveal that absenteeism decreased and Maslach Burnout Inventory scores increased, but scores on the Generalized Anxiety Disorder-7 (GAD-7) and Patient Health Questionnaire-9 (PHQ-9) significantly decreased. This implies that resilience and mental health at work have improved (65, 121). On the other hand, the studies’ small sample sizes, lack of long-term follow-up, and stark differences from one another make the evidence weak. More trustworthy randomized controlled trials (RCTs) are therefore required.

Tools with AI capabilities, such as Wysa, a chatbot, have shown promise (Table 2). Particularly during the COVID-19 pandemic, randomized controlled trials (RCTs) have demonstrated that they can improve depression scores and reduce anxiety symptoms by 60% (*p* = 0.004) among healthcare workers (122). These tools offer easily accessible and scalable support, but further study is required to verify their long-term impacts. With an effect size of d = 0.7, teletherapy—which encompasses both synchronous and asynchronous techniques—performs equally well as in-person cognitive behavioral therapy for anxiety and depression (123). Nurse-specific interventions such as the Resilience Hub demonstrated a 50% (120) reduction in burnout scores in a 2024 pilot study. However, it is difficult to determine how effective they are for everyone due to the small sample sizes. These findings suggest that, provided the methodological issues are resolved through larger-scale RCTs, DMHIs may be a versatile and successful means for nurses to receive mental health support.

**Table 2:** Comparison of digital mental health tools for nurses.

Tool type	Features	Effectiveness (evidence)	Limitations	Nurse relevance
Mobile apps	CBT, mindfulness, micro-interventions (5–10 min), gamification (119, 134)	Moderate–high effect size (d = 0.5–0.8) for anxiety/depression reduction (*p* < 0.01); ~80% satisfaction (65, 119)	High heterogeneity; low adherence (20–30%) (22, 139, 228)	Fits irregular shifts; reduces burnout/stress (65, 135)
AI tools	Chatbots, personalized feedback, 24/7 access (122, 141)	anxiety reduction (*p* = 0.004); improved depression scores (122, 126)	Limited long-term data; algorithmic bias, privacy risks (25, 141, 142)	Scalable, high engagement during pandemics (122)
Teletherapy	Online CBT, synchronous/asynchronous video or text sessions (123)	Effect size (d ≈ 0.7); comparable to in-person therapy (123)	Internet barriers, access issues in rural settings (125, 126)	Flexible, feasible for remote and overworked nurses (123)
Nurse-specific tools	Tailored resilience training and burnout programs (121, 134)	~50% burnout reduction (pilot studies, 2024); improved MBI scores (120, 121, 134)	Small samples; limited generalizability (120, 121)	Directly targets compassion fatigue and burnout (121, 134, 135)

Including key features, target mental health outcomes, level of evidence, accessibility, and limitations, based on recent literature (2020–2025). CBT, Cognitive Behavioral Therapy; MH, Mental Health; PTSD, Post-Traumatic Stress Disorder; AI, Artificial Intelligence; mHealth, Mobile Health; RCT, Randomized Controlled Trial; UI, User Interface; UX, User Experience.

### 5.2 Accessibility

Systemic, social, and technological factors affect nurses’ access to DMHIs. Since 85% (124) of nurses own smartphones, DMHIs are viable for widespread use because they offer round-the-clock access and typically have free or inexpensive alternatives. However, 20% of nurses lack reliable internet access in low-income and rural areas, making the digital divide a formidable obstacle (119, 125). Barriers in these settings are exacerbated by low technology adoption, poor connectivity, and the stigma associated with mental health care (22, 126). For instance, digital integration has improved mental health outcomes for low-income urban groups, but its efficacy is still limited in rural areas (120).

Language hurdles and cultural adaptation for minority and non-English speaking nurse populations must be addressed by DMHIs in order to increase equity (127). If technical difficulties and impersonality are lessened, rural nurses—who have less access to mental health specialists—will be able to use DMHIs (126, 128). Perceived utility, usability, and community engagement are adoption facilitators, particularly for senior nurses who may require specialized training (129, 130). Ethical access can be improved by strategies like funding support and mentoring for underrepresented nursing students, as well as models like PIDAR (Partner, Identify, Demonstrate, Access, Report) (131, 132). Making DMHIs work in a variety of settings requires overcoming these obstacles with scalable, user-centered designs.

### 5.3 Usability

Because user engagement has a direct impact on mental health, usability is a critical component of DMHI’s effectiveness. Short interventions (5–10 min), intuitive interfaces, and gamification components (such as story- or theme-based features) increase program retention and decrease dropout rates (119, 133). For nurses with erratic schedules, mobile and web-based interventions that emphasize sleep, exercise, and mental health—such as the Resilience Hub and Provider Resilience—are highly beneficial and pertinent. Eighty percent of trial participants reported satisfaction (121, 134, 135). However, depending on the duration of the intervention, the level of social support, and the user’s level of involvement in the design, adherence can vary greatly (28–100%) (123, 136).

Lack of time, feeling that it is inconvenient, and losing interest in the product are some factors that worsen usability. Conversely, real-time feedback and user-friendly interfaces are factors that improve usability (136, 137). According to some research, only 20–30% of users complete an app (138). This is a result of the app’s subpar design. Because many of the studies that are currently being conducted use small sample sizes and single-questionnaire assessments, which make it difficult to generalize, a systematic review revealed that we need standardized methods to test usability (139). Working with nurses to ensure that DMHIs have individualized content and effective ways to engage people is crucial to maximizing their effectiveness in high-stress healthcare settings.

### 5.4 Security and privacy

Nurses’ acceptance and confidence in DMHIs depend heavily on privacy and security, particularly as these systems integrate with AI-powered technologies and EHRs. Threats of unauthorized data access, a lack of security measures, and insufficient training are frequently cited by nurses as challenges (23). Only 25% of healthcare apps explicitly mentioned HIPAA compliance, 18% mentioned GDPR, and 79% lacked defined data breach procedures, according to a review (24). Trust is also damaged by discrepancies between stated privacy policies and actual compliance with laws like the CCPA and GDPR (140).

While AI-powered DMHIs hold promise for reducing workloads and supporting mental health, they also raise issues with algorithmic bias and ethical integration (141, 142). Although third-party data sharing and opaque AI algorithms remain major concerns, nurses value discrete tools that align with therapeutic goals to prevent stigma in the workplace (25). Healthcare organizations must implement strict data protection policies, enhance informatics education, and encourage ethical adherence in order to meet these demands (23, 143). In order to maximize the use of these tools while maintaining security and privacy, as well as to ensure trust and human-centered care, nurses must be included as co-designers of DMHIs.

### 5.5 Critical analysis and gaps

Despite their potential, DMHIs face significant barriers to widespread adoption. Research is often limited by small, non-diverse samples, which limits generalizability, but peer group interventions and individual digital programs demonstrate efficacy in reducing stress and fostering resilience (144, 145). Too many interventions are still in pilot stages, and the absence of longitudinal data obscures long-term effects (146, 147). Despite their cost-effectiveness, algorithmic bias, data confidentiality, and lack of integration within healthcare systems are issues that plague AI-powered DMHIs and can undermine trust (18, 141). The modest effects of workplace digital solutions on work engagement point to the need for interventions that focus on particular nursing stressors, such as spiritual exhaustion and moral injury.

Large-scale, varied studies with extended follow-up periods should be given priority in order to advance the field because they offer generalizability and long-term efficacy (147). In order to address AI-related concerns like bias and patient autonomy and to ensure equitable use of DMHIs, nursing-specific ethical standards must be developed (25, 148). Additionally, inclusive research environments must be promoted. These are essential to achieving DMHIs’ full potential in supporting nurses’ mental health.

With moderate to strong evidence demonstrating their effectiveness in lowering depression, anxiety, and burnout, DMHIs provide scalable and efficient solutions for nurses’ mental health issues. However, obstacles like the digital divide, privacy issues, usability issues, and a lack of longitudinal data prevent them from being widely adopted. Through the prioritization of user-centered designs, strong privacy protocols, and inclusive research, DMHIs can better address the varied needs of nurses, improving workplace resilience and mental health in high-stress healthcare environments.

## 6 Application in nursing practice

DMHIs are changing how nurses deal with trauma, stress, and resilience in the context of the changing healthcare environment. These cutting-edge resources, which include wearable technology and smartphone apps, are becoming essential in clinical and professional settings and provide real-time support for nurses’ mental health. This section explores the clinical applications, integration into workplace mental health programs, ethical issues, and the crucial role nurses played in the development and adoption of DMHIs. It also offers a structured implementation pathway.

### 6.1 Integration in clinical practice

DMHIs are becoming essential tools to address the psychological demands that nurses face in high-pressure clinical settings. Through interventions designed for shift breaks, mobile apps such as Headspace, which offer quick mindfulness exercises, have demonstrated efficacy in lowering stress and burnout (149, 150). Short meditation sessions have been shown to improve attention and reduce stress, and research shows that they can improve psychological detachment, mood, and well-being (70, 151). After an incident, teletherapy, especially cognitive-behavioral therapy (CBT) administered through telehealth, provides strong support by reducing symptoms of trauma and improving professional functioning. Nurse-led CBT interventions are as effective as those conducted by mental health professionals (152). Randomized controlled trials have shown that resilience training has significantly reduced stress and anxiety while promoting resilience among intensive care unit (ICU) nurses. This includes multimodal programs that combine workshops and counseling, as well as mobile-based micro-learning (153, 154).

**Figure 3:**
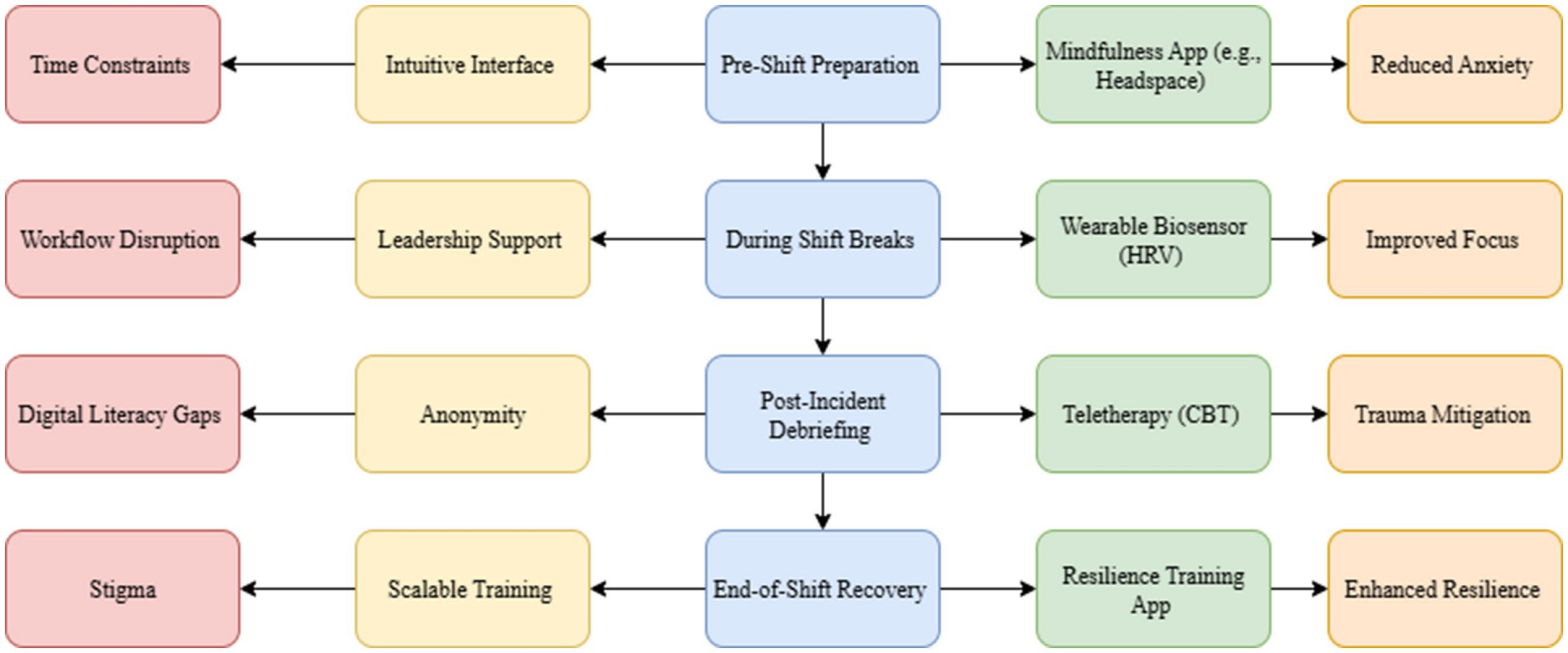
Conceptual framework illustrating the integration of digital mental health interventions (DMHIs) into the nursing workflow to address psychological demands in high-pressure clinical settings. The diagram maps common barriers—such as time constraints, workflow disruption, digital literacy gaps, and stigma—to corresponding enabling strategies (e.g., intuitive interface, leadership support, anonymity, and scalable training). These strategies are linked to context-specific intervention points across the nursing shift, including pre-shift preparation, during-shift breaks, post-incident debriefing, and end-of-shift recovery. Examples of targeted DMHIs include mindfulness applications (e.g., Headspace), wearable biosensors for heart rate variability (HRV) monitoring, teletherapy using CBT, and resilience training applications. Each intervention is connected to evidence-based psychological outcomes such as reduced anxiety, improved focus, trauma mitigation, and enhanced resilience.

Real-time stress monitoring is made possible by wearable technology, such as wrist-worn biosensors that use heart rate variability (HRV) analysis. This allows for customized mindfulness interventions to improve emotional self-regulation (111, 155). Time constraints and the integration of DMHIs into clinical workflows continue to be obstacles despite these developments (150, 151) (Figure 3). For DMHIs to be consistently adopted and to have the most significant possible impact across a range of clinical settings, these obstacles must be addressed structurally through improvements like simplified workflow integration.

### 6.2 Programs for workplace mental health

Fostering resilience and lowering occupational stressors are largely dependent on workplace initiatives that support nurses’ mental health. Effective DMHI integration is demonstrated by programs like employee assistance programs (EAPs) and hospital wellness initiatives like CopeNYP at NewYork-Presbyterian Hospital, which offers digital short-term counseling to over 38,000 employees with a 4.25% utilization rate, resulting in fewer therapy sessions and better emotional outcomes (102, 156). Notable advantages of digital peer support in EAPs have also been observed, such as a 15% decrease in turnover in pilot programs and a positive social return on investment, highlighting the effectiveness of these programs in improving nurse retention (156).

Adoption of DMHI is greatly aided by nurse managers, who act as educators and advocates to lessen the stigma associated with mental health care (157). However, managers’ high levels of anxiety and lack of training in digital health competencies underscore the necessity of organized mentoring and empowerment education (158, 159). To successfully integrate DMHIs and foster a positive corporate culture, managers must be proficient in data literacy and health informatics (160, 161). To guarantee sustainable DMHI implementation and eventually enhance nurse retention and patient care outcomes, future initiatives should concentrate on scalable training and stigma-reduction techniques.

### 6.3 Ethical considerations

In order to implement AI-driven DMHIs in a fair and responsible manner, it is imperative that ethical issues be addressed. Clear communication regarding these tools’ clinical utility, evidence base, accuracy, data security, and impact on autonomy is necessary for informed consent (162). Vulnerabilities in mobile app security, like poor permission modeling and vulnerability to cyberattacks, exacerbate privacy concerns and call for strong data protection measures (163). Collaboration between nurses and developers is necessary to ensure fairness and foster trust because algorithmic biases in natural language processing models, particularly those pertaining to race, gender, and culture, run the risk of escalating health disparities (164). It is important to strike a balance between technology and human connection because nurses are concerned that digital tools could depersonalize care and undermine professional identity. Maintaining the compassionate nature of nursing while addressing these ethical issues requires a human-centered approach that involves nurses in the development and continuous assessment of AI tools (165, 166).

### 6.4 Nurse participation

To guarantee that DMHIs are useful, pertinent, and easily incorporated into clinical practice, nurses must be involved in their creation and application. By allowing nurses to influence tool functionalities, co-design methodologies like user-centered design (UCD) and participatory design (PD) produce user-friendly interfaces and increased engagement (167, 168). Co-designed visual handover aids, for example, have facilitated information access, decreased navigation time, and promoted long-lasting enhancements in nursing practices (169, 170). However, obstacles like high development costs and workload demands can make it difficult for nurses to participate, so strong engagement tactics are needed to keep them involved (171, 172). Another issue is digital literacy, as about 30% of senior nurses say they are not confident using technology (173). Nurses’ abilities and perspectives on mobile health applications have improved as a result of focused interventions like virtual training and sessions led by librarians (174, 175). To enhance nurse participation, structured training modules—developed collaboratively with nurses and drawing from implementation science—can include key components such as familiarization with DMHIs, evidence from randomized controlled trials, referral processes, scripted patient presentations, data interpretation, and workflow integration (176). These modules should be delivered iteratively, incorporating peer-to-peer sharing and refreshers in routine clinic meetings to build competency and confidence. To improve DMHI effectiveness and prepare nurses for digital healthcare environments, nursing curricula must incorporate digital literacy competencies that cover information technology, health informatics, and data privacy (177, 178). Furthermore, to overcome resistance to participation, incentives such as continuing education credits, recognition of nurse champions who lead co-design efforts, and integration of brief training into existing workflows (e.g., with rewards like catered sessions) can foster buy-in and sustained involvement, aligning with actionable insights from implementation science frameworks like RE-AIM for evaluating reach and adoption (176).

**Figure 4:**
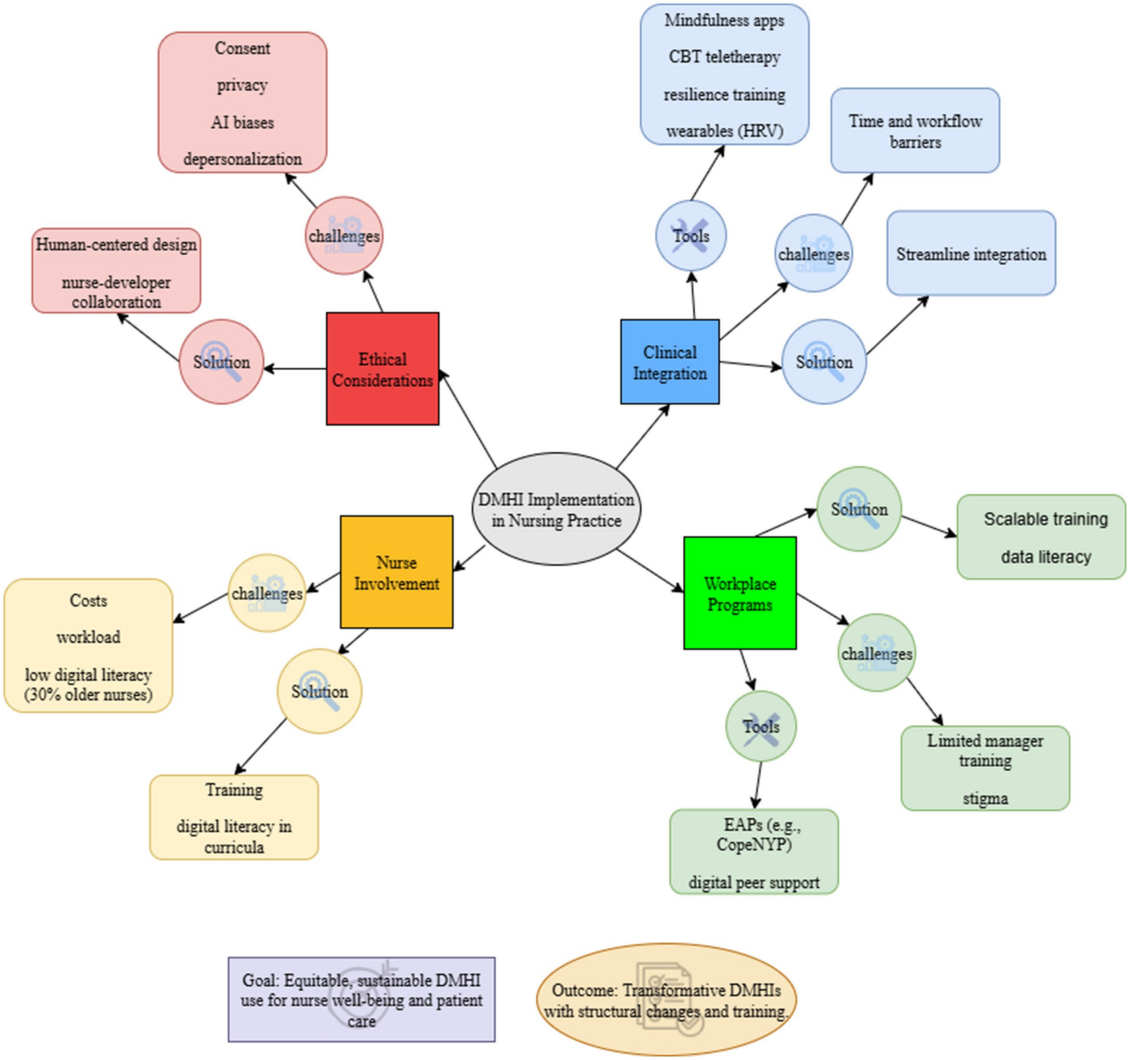
A framework for integrating digital mental health interventions (DMHIs) into nursing practice that emphasizes four interrelated areas: workplace programs, clinical integration, ethical considerations, and nurse involvement. Every domain lists the main issues (such as privacy issues, AI biases, workflow obstacles, low digital literacy, and stigma) and their corresponding fixes (such as scalable training, human-centered design, streamlined integration, and integrating digital literacy into nursing curricula). Employee assistance programs (EAPs), digital peer support platforms, wearable biosensors for HRV monitoring, mindfulness applications, CBT-based teletherapy, and resilience training programs are a few examples of tools. The model highlights how tackling these issues with focused tactics can result in fair, long-lasting DMHI adoption, which will enhance nurse well-being and patient care results.

Ultimately, DMHIs have the capacity to significantly improve nursing professionals’ mental health, resilience, and occupational stress management. While it is essential to integrate them into clinical practice, workplace programs, and co-design processes, there are several obstacles to overcome, including ethical dilemmas, time restraints, and gaps in digital literacy (Figure 4). Prioritizing human-centered design, scalable training, and structural changes will help healthcare systems implement DMHI in a fair and sustainable manner, improving patient care outcomes and nurse well-being.

## 7 Difficulties and obstacles

Using cutting-edge technologies like machine learning and DMHIs, artificial intelligence (AI) has the potential to revolutionize hospital electronic health record (EHR) systems and mental health nursing by facilitating improved patient care and promoting nurse well-being (19, 93, 179–181). However, there are many obstacles to overcome before incorporating AI into clinical practice. These include technological constraints like low-quality data and interoperability problems (182, 183), ethical and legal issues about algorithmic bias and data privacy (18, 184), adoption barriers brought on by nurses’ lack of training and worries about dehumanizing care (185, 186), and equity issues brought on by the digital divide and a lack of cultural sensitivity (187, 188). To address these, practical recommendations from implementation science include staged implementation approaches: beginning with pilots (e.g., testing with 25–50 nurse referrals, tracking key performance indicators like activation rates and satisfaction), progressing to scaling (expanding to more sites with checkpoints), and ensuring sustainability through ongoing training refreshers and decision gates. Continuous quality improvement (CQI) using Plan-Do-Study-Act (PDSA) cycles can iteratively resolve difficulties, such as low uptake, by automating referrals via EHRs and employing digital navigators for patient follow-up within 24–48 h. Incentives to overcome resistance might involve leveraging nurse champions for peer feedback, providing regular success metrics (e.g., symptom reduction via PHQ-9 scores), and offering tangible rewards like continuing education credits to build trust and reduce negativity bias (176). In order to promote fair, moral, and efficient AI implementation and improve mental health outcomes, this section critically analyzes these complex issues and suggests evidence-based solutions (Table 3).

**Table 3:** Challenges and solutions for digital mental health intervention (DMHI) adoption in nursing.

Challenge type	Description	Impact	Solutions
Technological	Poor data quality (incomplete, inaccurate, or biased datasets) (182); interoperability issues between mobile mental health apps and EHR/EMR systems (183); lack of standardized evaluation frameworks for AI tools (189).	Compromises reliability of AI algorithms leading to incorrect assessments and unfavorable clinical decisions (182); restricts integration and use in psychiatric treatment (183); complicates evaluation of effectiveness and dependability (189).	Implement algorithmic auditing to reduce biases and ensure data integrity (93); adopt Fast Healthcare Interoperability Resources (FHIR) standards for system compatibility (93); prioritize high-quality data and interoperable systems to automate tasks and improve patient outcomes (141, 190).
Ethical	Opacity in AI decision-making processes (e.g., non-explainable AI) (191); algorithmic bias from biased or unrepresentative training data (186, 193); lack of transparency, accountability, and trust (193); regulatory ambiguity and insufficient nursing-specific guidelines (18, 184, 194); concerns over data privacy and informed consent (18, 192).	Undermines patient autonomy and informed consent (192); potential for harm in critical mental health support (184); perpetuates unfair care outcomes (186); erodes trust in clinical settings (193).	Develop nursing-specific ethical guidelines and oversight mechanisms (18, 194); apply bias mitigation techniques and continual assessments (186, 193); utilize frameworks like FAITA-Mental Health for transparency and stakeholder engagement (192, 193); foster collaboration between nurses, AI developers, and regulators (192, 193); embed principles like privacy, informed consent, and culturally sensitive design in AI development (186).
Adoption	Inadequate training and lack of experience with AI among nurses (185); resistance due to discomfort, fear of replacing human interaction, and concerns over biases or depersonalization (185, 186); organizational factors like staffing shortages, time constraints, and workflow disruptions (195, 196); lack of user-centered design in mental health apps (e.g., poor navigation, non-personalized content) (135).	Leads to reluctance and resistance to AI adoption (185); exacerbates challenges in high-pressure clinical environments (195, 196); results in low engagement and long-term use of tools (135); hinders effective integration into nursing practice (186, 195).	Create comprehensive AI education programs tailored for nurses (186, 195); involve nursing staff early in AI tool design and development (186, 195); promote interdisciplinary cooperation between AI developers and mental health professionals (186, 195); provide organizational support including clear implementation policies, technology access, and dedicated support staff (197, 198).
Equity	Digital divide limiting access in resource-constrained settings (e.g., workload pressures, low digital proficiency, limited computer access) (187); barriers for marginalized groups including financial constraints, lack of culturally appropriate resources, and social isolation (125); risk of AI tools reinforcing biases without cultural adaptation (188, 200); high attrition rates and lower technology ownership in underserved/rural areas (126, 202).	Exacerbates mental health disparities among diverse and underserved populations (125, 187); leads to unfair care outcomes and disproportionate impacts on minorities (188, 200); hampers sustained engagement and access to mental health support (126, 202); widens inequities in healthcare delivery (187, 188).	Encourage stakeholder collaboration in tool development (203, 204); incorporate culturally sensitive design (188, 200, 201); provide thorough digital literacy training for nurses (187, 203); emphasize cultural competence to close the digital divide (65, 188, 201); promote inclusive strategies to enhance access and adoption across diverse populations (203, 204).

A systematic overview of technological, ethical, adoption, and equity barriers to integrating AI-driven digital mental health interventions, with evidence-based solutions to promote effective and equitable implementation. HER, Electronic Health Record; EMR, Electronic Medical Record; AI, Artificial Intelligence; FHIR, Fast Healthcare Interoperability Resources; NXAI, Non-Explainable Artificial Intelligence; GDPR, General Data Protection Regulation; FAITA-Mental Health, Framework for AI Tool Assessment in Mental Health.

### 7.1 Technological difficulties in integrating AI with mental health nursing

Significant technological obstacles stand in the way of the integration of artificial intelligence (AI) into hospital electronic health record (EHR) systems and mental health nursing programs, especially with regard to data quality and system interoperability. The dependability of AI algorithms in digital mental health tools is jeopardized by poor data quality, which is defined by partial, erroneous, or biased datasets. This could result in incorrect assessments and unfavorable clinical decisions (182). This problem is particularly noticeable in mental health nursing, where mental health conditions are assessed and diagnosed using AI tools like machine learning and natural language processing (19). A major barrier is also the absence of smooth communication between mobile mental health apps and current EHR systems, since these apps frequently do not work well with clinical processes and electronic medical records (EMRs), which restricts their use in psychiatric treatment (183). The evaluation of the effectiveness and dependability of these applications is made more difficult by the lack of standardized evaluation frameworks (189). Strong approaches are required to overcome these obstacles, such as implementing algorithmic auditing to reduce biases and guarantee data integrity and adopting Fast Healthcare Interoperability Resources (FHIR) standards to enhance system compatibility (93). As long as high-quality data and interoperable systems are given priority, overcoming these technological obstacles is essential to utilizing AI’s potential to improve mental health nursing by automating repetitive tasks and improving patient outcomes (141, 190).

### 7.2 Regulatory and ethical difficulties in AI for mental health nursing

Artificial intelligence (AI)-powered mental health interventions pose difficult ethical and legal issues that must be carefully considered in order to be implemented responsibly in nursing practice. Concerns about accountability, trust, and transparency in clinical settings are brought up by the opacity of AI decision-making processes, especially with non-explainable AI (NXAI) (191). Patients may not fully understand how AI-generated recommendations are derived, which could compromise their autonomy and undermine informed consent (192). Additionally, biased or unrepresentative training data can lead to algorithmic bias in mental health applications, which calls for strong bias mitigation techniques and continual assessment (186, 193). The need for nursing-specific ethical guidelines and oversight mechanisms is highlighted by the fact that, although regulatory frameworks such as the General Data Protection Regulation (GDPR) offer a foundation for data protection, they frequently lack specificity for nursing contexts (18, 194). Numerous AI-enabled wellness applications are subject to regulatory ambiguity, which makes it difficult to integrate them safely. This is because these tools are often not thoroughly evaluated, which raises the possibility of harm in situations involving critical mental health support (184). Frameworks like the Framework for AI Tool Assessment in Mental Health (FAITA-Mental Health) support transparency, reproducibility, and stakeholder engagement in order to tackle these issues. They also encourage cooperation between mental health nurses, AI developers, and regulators in order to put patient safety and equity first (192, 193). In order to promote person-centered care, mental health nurses should take the lead in developing these technologies and making sure that ethical concepts like privacy, informed consent, and culturally sensitive design are ingrained in AI development (186).

### 7.3 Adoption barriers in AI for mental health nursing

Significant obstacles that stem from organizational and individual factors prevent nurses from implementing AI-driven digital mental health tools and integrating them into clinical practice. Inadequate training and nurses’ lack of experience with AI technologies pose a significant obstacle, leading to resistance because of discomfort and worries that AI could replace human interaction in the provision of care (185). Reluctance is further exacerbated by nurses’ preference for a humanistic approach and their concern that AI systems may introduce biases or depersonalize patient interactions (186). As nurses deal with time constraints and workflow disruptions in high-pressure clinical settings, organizational challenges like staffing shortages and inadequate support during technology implementation make these problems worse (195, 196). Furthermore, a lot of mental health apps lack user-centered design elements that are necessary for engagement and long-term use, like personalized content and easy navigation (135). Targeted approaches are needed to overcome these obstacles, such as creating thorough AI education programs specifically for nurses, involving nursing staff early in the design and development of AI tools, and encouraging interdisciplinary cooperation between AI developers and mental health professionals to match tools with clinical requirements (186, 195). Building nurse confidence and promoting successful adoption also requires organizational support, which includes things like clear implementation policies, technology access, and committed support staff (197, 198). AI technologies can help nurses provide effective, high-quality mental health care while upholding the person-centered philosophy of nursing practice by removing these obstacles.

### 7.4 Cultural and ethical aspects of AI in mental health nursing

Although there is great promise for addressing mental health disparities through the integration of AI-driven DMHIs into nursing practice, equity and cultural barriers present challenges, especially for underserved and diverse populations. In resource-constrained settings, where workload pressures, limited computer access, and low digital proficiency pose significant obstacles, nurses’ access to these tools is severely limited by the digital divide (187). Racial and ethnic minorities, among other marginalized groups, face additional obstacles like financial limitations and a lack of culturally appropriate resources, and these difficulties are exacerbated by social isolation during the COVID-19 pandemic (125, 199). Because unadapted AI tools run the risk of reinforcing biases that disproportionately affect minority populations and could result in unfair care outcomes, cultural competence is crucial for equitable mental health care (188, 200). Mental health applications (MHAs) designed for diverse nursing populations, for instance, have been shown to be effective in lowering anxiety and burnout, with interventions that are culturally appropriate having a noticeably higher impact (65, 201). DMHIs can help reduce stigma and clinician access barriers in underserved and rural areas where mental health issues are common, but sustained engagement is hampered by high attrition rates and lower technology ownership (126, 202). Strategies like stakeholder collaboration in tool development, culturally sensitive design, and thorough digital literacy training for nurses are essential to addressing these issues and fostering equitable access and adoption (203, 204). AI-driven DMHIs can improve mental health support for nurses and patients by emphasizing cultural competence and closing the digital divide, promoting inclusive and efficient care delivery across a range of populations.

## 8 Recommendations for future development and research

**Figure 5:**
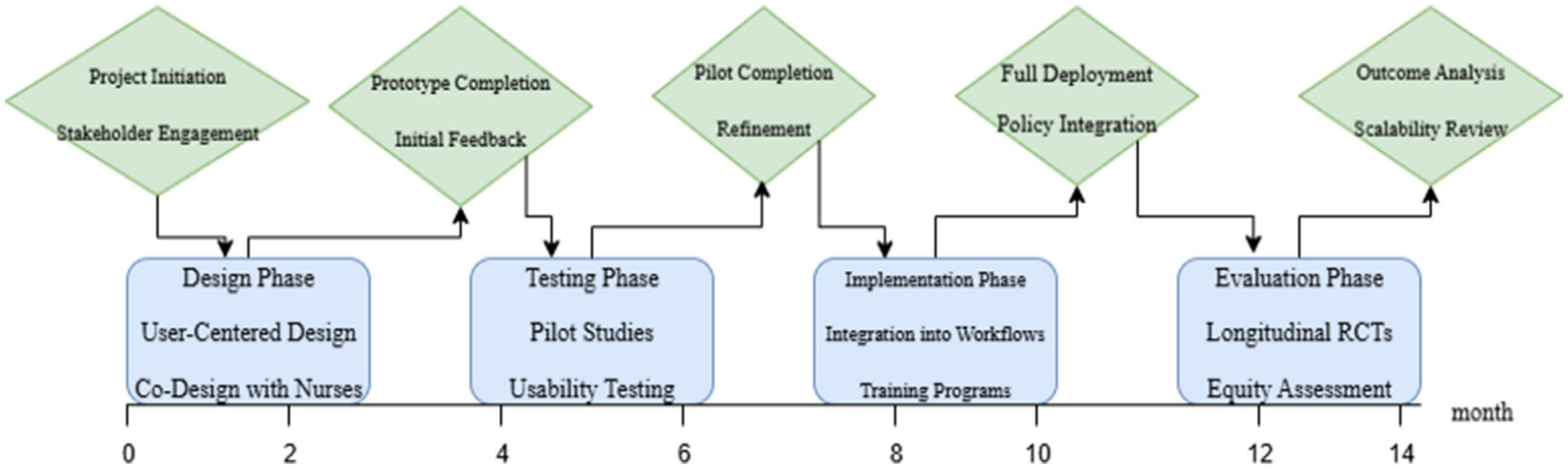
Strategic plan for nurse-focused digital mental health interventions (DMHIs). An estimated 14 months would be needed for the design, testing, implementation, and evaluation phases of the suggested development and implementation timeline for DMHIs in nursing practice. Project initiation, stakeholder engagement, and co-design with nurses utilizing user-centered design principles are all part of the Design Phase, which lasts between 0 and 2 months. Pilot studies, usability testing, prototype completion, and preliminary feedback are all included in the testing phase, which lasts two to six months. The pilot’s completion, improvement, full deployment, integration into clinical workflows, and training initiatives are all included in the six to ten-month Implementation Phase. Outcome analysis, policy integration, longitudinal randomized controlled trials (RCTs), equity assessment, and scalability review are all part of the evaluation phase, which lasts 10–14 months. In order to enhance patient care and mental health outcomes, this phased approach guarantees that DMHIs are thoroughly tested, contextually adjusted, and sustainably incorporated into nursing practice.

DMHIs present a promising way to address the particular stressors encountered in nursing practice, such as shift work stress, trauma, and moral distress, as the mental health issues of nurses receive more and more attention. Future initiatives must focus on developing nurse-specific tools, expanding research to assess long-term efficacy and equity, and establishing robust policy frameworks to support moral and long-term implementation, if they are to have the greatest possible impact. A thorough plan for developing DMHIs is presented in this section, with a focus on strategic policy and practice initiatives, rigorous research, and customized development to improve nurse well-being in a range of healthcare environments (Figure 5).

### 8.1 Progress

Customized DMHIs that focus on stressors like shift work, trauma, and moral distress are necessary to address the psychological demands of nursing. A randomized controlled trial has demonstrated the effectiveness of programs like the SleepCare cognitive behavioral therapy initiative in reducing insomnia in nurses with shift work sleep disorder (205). Similarly, in high-stress environments such as intensive care units (ICUs) and emergency rooms, internet-based interventions, such as interactive websites and smartphone apps, have reduced stress, anxiety, and depression (206). Moral distress, particularly among critical care nurses, remains poorly understood. Although some research suggests that empowerment initiatives and educational workshops may be beneficial, further research is needed (207).

User-centered design concepts, like offline mental health literacy apps and content catered to nurses’ unique stressors, should be incorporated into future DMHIs (135, 208). Delivered through smartphones and utilizing real-time ecological momentary assessments (EMAs), ecological momentary interventions (EMIs) provide just-in-time assistance to encourage resilience and healthy habits (209). In addition to lowering workload and stress, features like digital task management tools, access to private respite spaces, and non-institutional aesthetics can also create supportive environments (210). Advanced encryption and user-controlled data-sharing options are crucial for privacy (165). Such tools will become increasingly relevant and sustainable across nursing specialties as they are further developed and combined with wearables for real-time monitoring.

### 8.2 Investigation

Future research must give priority to longitudinal randomized controlled trials (RCTs), nurse-focused studies, equity considerations, and implementation science in order to develop a substantial body of evidence for DMHIs. Given the conflicting results of previous studies, longitudinal RCTs lasting 12–24 months are essential for evaluating long-term outcomes like burnout reduction. During the COVID-19 pandemic, for example, some longitudinal studies found no significant differences in mental health outcomes between intervention and control groups. In contrast, others observed both positive and negative changes, with supportive environments being associated with better well-being (211, 212). In order to address setting-specific stressors like heavy workloads and scarce resources, nurse-focused studies should investigate a variety of settings, including intensive care units, rural hospitals, and community health. Although digital interventions have demonstrated moderate to significant effects on mental health literacy and high acceptability in intensive care units, more research is needed to determine their cost-effectiveness and cultural diversity (213, 214).

To guarantee that DMHIs lessen disparities for low-resource and minority nurses, equity-focused research is essential. Though more widespread validation is required, tactics like bilingual options and AI-enabled chatbots have improved engagement among marginalized groups (215, 216). By identifying organizational facilitators like leadership engagement and absorptive capacity, implementation science can improve real-world adoption through the use of frameworks like CFIR (Consolidated Framework for Implementation Research) and RE-AIM (Reach, Effectiveness, Adoption, Implementation, Maintenance) (217, 218). The creation of efficient, fair, and scalable DMHIs suited to various nursing contexts will be guided by these research priorities.

### 8.3 Practice and policy

Strong policy frameworks and workable tactics are necessary for the successful adoption and impact of DMHIs in nursing practice. The development and application of digital tools, which have proven effective in addressing stress and burnout through low-threshold access, care navigation, and evidence-based online psychotherapy, require sustained funding (14, 219, 220). Digital platform-based employer-sponsored programs improve access to high-quality mental health services and provide affordable options for nurses in a variety of specialties (82, 219). However, economic assessments point out that more research is necessary to determine long-term cost-effectiveness in comparison to conventional interventions, such as in-person therapy (221, 222).

Privacy protection, informed consent, and bias mitigation must be given top priority in ethical guidelines for AI-driven DMHIs, especially in nursing contexts where AI increases efficiency but raises questions about autonomy and transparency (18, 223). Actionable insights from implementation science include stakeholder mapping to engage nurses, leadership, and IT teams early; establishing key performance indicators (KPIs) such as referral rates, equity measures (e.g., engagement among underserved nurses), and symptom reduction; and using CQI processes to refine policies iteratively. For practice, incentives like recognition of champions and integration of DMHIs as frontline tools in primary care pathways can overcome resistance, while training modules with scripted referrals and automated EHR processes enhance adoption (176). To develop user-centered tools that are suited to a variety of needs, including those of culturally and linguistically diverse communities, co-design collaboration between nurses, developers, and policymakers is crucial (224, 225). As demonstrated by effective nurse home visiting programs, multi-stakeholder partnerships across the health, education, and technology sectors can further encourage adoption by addressing organizational barriers and fostering competency development (226, 227). To ensure sustainable and equitable DMHI implementation, these initiatives highlight the necessity of inclusive co-design, strict ethical frameworks, and scalable funding models.

## 9 Conclusion

The escalating mental health challenges faced by nurses require solutions that are adaptable, sustainable, and capable of addressing both systemic and individual barriers to care. DMHIs have demonstrated encouraging outcomes in reducing burnout, anxiety, depression, and post-traumatic stress disorder while promoting resilience, job satisfaction, and retention. These include mobile health applications, AI-driven support tools, teletherapy, and hybrid platforms. These advantages are especially pertinent in healthcare settings where stigma, time restraints, and a lack of staff restrict access to conventional support services.

Critical limitations are also revealed by the evidence, however. The broad adoption and sustained efficacy of DMHIs are impeded by disparities in access brought about by the digital divide, disparities in digital literacy, privacy and data security issues, and a lack of smooth integration into clinical workflows. The lack of extensive, long-term, nurse-specific research also restricts the applicability of existing findings, highlighting the necessity of thorough studies that represent a range of nursing contexts.

Co-designing DMHIs with nurses is essential to maximizing their impact because it ensures cultural relevance, usability, and alignment with clinical realities like high-stress specializations and irregular shifts. Transparency, bias reduction, and informed consent should be given top priority in ethical frameworks, especially when it comes to AI-enabled tools. DMHIs should be incorporated into larger workplace wellness initiatives as part of implementation strategies, which should be backed by organizational policies that encourage psychological safety, leadership involvement, and ongoing training.

To reinforce the transformative potential of DMHIs and address evidence-based gaps, future research priorities must include large-scale randomized controlled trials (RCTs) to rigorously evaluate long-term efficacy, cost-effectiveness, and scalability across diverse nursing populations, including underrepresented groups in rural and low-resource settings. Additionally, policy implications extend to synthesizing practice guidelines for seamless DMHI integration into healthcare systems, such as mandating funding for digital literacy programs, establishing regulatory standards for privacy and equity, and incentivizing multi-stakeholder collaborations to mitigate disparities and enhance overall impact on nurse well-being and patient care.

In the end, a cooperative strategy involving healthcare professionals, legislators, researchers, and technology developers is necessary for the effective integration of DMHIs into nursing practice. Healthcare systems can establish a setting where mental health support is not only available and tailored to each individual, but also integrated into the routine nursing practice by utilizing both human empathy and digital innovation. A more sustainable healthcare system, better patient outcomes, and a more resilient nursing workforce are all possible with this strategy.
